# Exogenous Hydrogen Sulfide Supplement Attenuates Isoproterenol-Induced Myocardial Hypertrophy in a Sirtuin 3-Dependent Manner

**DOI:** 10.1155/2018/9396089

**Published:** 2018-12-17

**Authors:** Jingyao Zhang, Jin Yu, Yun Chen, Lulu Liu, Mengting Xu, Linlin Sun, Huiqin Luo, Yuqin Wang, Guoliang Meng

**Affiliations:** ^1^Department of Pharmacology, School of Pharmacy, Nantong University, Key Laboratory of Inflammation and Molecular Drug Target of Jiangsu Province, Nantong, 226001 Jiangsu, China; ^2^Department of Pharmacology, Yancheng City No. 1 People's Hospital, Yancheng, 224001 Jiangsu, China

## Abstract

Hydrogen sulfide (H_2_S) is a gasotransmitter with a variety of cardiovascular protective effects. Sirtuin 3 (SIRT3) is closely related to mitochondrial function and oxidative stress. We found that NaHS increased SIRT3 expression in the preventive effect on isoproterenol- (ISO-) induced myocardial hypertrophy. We further investigated whether exogenous H_2_S supplement improved ISO-induced myocardial hypertrophy in a SIRT3-dependent manner. 10-week-old male 129S1/SvImJ (WT) mice and SIRT3 knockout (KO) mice were intraperitoneally injected with NaHS (50 *μ*mol/kg/d) for two weeks and then intraperitoneally injected with ISO (60 mg/kg/d) for another two weeks. In WT mice, NaHS significantly reduced the cardiac index of ISO-induced mice, decreased the cross-sectional area of cardiomyocytes, and inhibited the expressions of atrial natriuretic peptide (ANP) and brain natriuretic peptide (BNP) mRNA. The activity of total antioxidant capacity (T-AOC) and superoxide dismutase (SOD) in the myocardium was increased, but the level of malondialdehyde (MDA) was decreased. The fluorescence intensity of dihydroethidium staining for superoxide anion was attenuated. Optic atrophy 1 (OPA1) expression was upregulated, while dynamin-related protein 1 (DRP1) expression was downregulated. ERK, but not P38 and JNK, phosphorylation was downregulated. However, all above protective effects were unavailable in ISO-induced SIRT3 KO mice. Our present study suggested that exogenous H_2_S supplement inhibited ISO-induced cardiac hypertrophy depending on SIRT3, which might be associated with antioxidant stress.

## 1. Introduction

Myocardial hypertrophy is a compensatory process with increased cardiac mass and myocardial contractility under a long-term pressure overload, which is beneficial to maintaining normal blood circulation [1]. However, there is no adequate coronary artery blood to meet the needs of the heart because hypertrophic myocardium requires more oxygen. It is easy to result in myocardial ischemia, myocardial contractility impairment, and eventually heart failure if the heart suffers from persistent pathological hypertrophy [2]. Multilevel and complex factors, such as oxidative stress, energy metabolism disorder, hemodynamic factors, neurohumoral factors, cardiovascular autocrine/paracrine factors, insulin resistance, microRNAs, and genetics, are involved in the progress of myocardial hypertrophy [3–9]. Until now, the exact molecular mechanism for myocardial hypertrophy is still incompletely clarified. It is important to find novel compounds to delay or reverse myocardial hypertrophy.

In the traditional concept, hydrogen sulfide (H_2_S) is a gas with highly toxic, which damages physiological functions [10]. A small amount of high concentration of H_2_S might achieve a fatal effect in a short time after inhalation [11]. But with the deepening understanding of H_2_S in recent years, it has revealed that H_2_S has important physiological functions in the nervous system, the immune system, the digestive system, and so on [12–17]. H_2_S widely exists in many mammals and is widely concerned as the third gasotransmitter followed by nitric oxide and carbon monoxide [18]. H_2_S is mainly produced by three enzymes: cystathionine *γ*-lyase (CSE), cystathionine *β*-synthase (CBS), and 3-mercaptopyruvate sulfurtransferase (MPST) [19].

Our previous studies found that H_2_S decreased blood pressure in spontaneously hypertensive rats (SHR) [20] and transverse aortic constricted mice [21], inhibited myocardial fibrosis in SHR [22], improved vascular endothelial function [23], and prevented diabetic atherosclerosis [24]. It suggested that H_2_S played an important role in cardiovascular system. However, the mechanisms of H_2_S on cardiac hypertrophy were not completely clear. Glucose-6-phosphate dehydrogenase [25], miR-133a upregulation [26], nuclear factor E2-related factor 2 (Nrf2) activation [27], and specific protein 1 S-sulfhydration [20] were previously thought to be the principal reasons for the protective effect of H_2_S on myocardial hypertrophy. These studies suggested that the detailed mechanisms for the effects of H_2_S on myocardial hypertrophy in different models were not completely consistent. Therefore, it is vital to explore the specific mechanism of H_2_S for delaying or reversing myocardial hypertrophy.

Sirtuin 3 (SIRT3) is a histone deacetylase which was encoded by the SIRT3 gene and localized in the mitochondria [28]. It is found that SIRT3 deficiency promoted cardiac fibrosis and left ventricular dilatation, which might even deteriorate to decompensated cardiac dysfunction in the aging mice [29]. In the rodent model of heart failure, SIRT3 expression and activity were decreased. Levels of mitochondrial protein lysine acetylation were increased, which might promote oxidative stress [30]. Impaired SIRT3 blocked fatty acid oxidation, glucose oxidation, tricarboxylic acid cycle, and oxidative phosphorylation, which might be a potential mechanism for contraction dysfunction and adaptation impairment [31–33]. Contrarily, SIRT3 protected the heart from reactive oxygen species- (ROS-) induced oxidative damage. Different pharmacological approaches to increase SIRT3 expression or to enhance SIRT3 activity were a potent strategy to attenuate myocardial hypertrophy and other cardiovascular diseases [34–38]. Our previous research also found that H_2_S increased SIRT3 promoter activity and SIRT3 expression in angiotensin II-induced cardiomyocyte hypertrophy [21]. However, the detailed information about the potential role of SIRT3 on myocardial hypertrophy by H_2_S *in vivo* was not known well.

As well as we know, dynamin-related protein 1 (DRP1) is a mitochondrial fission-associated protein, while optic atrophy 1 (OPA1) is a mitochondrial fusion-associated protein [39–42]. The balance of DRP1 and OPA1 plays an important role in maintaining mitochondrial structure and function. However, whether H_2_S regulated DRP1 and OPA1 in the protective effect against myocardial hypertrophy was unknown.

The aims of the present study were to examine whether exogenous H_2_S supplement attenuated isoproterenol- (ISO-) induced myocardial hypertrophy in SIRT3 knockout (KO) mice. It is beneficial to elucidate the possible role of SIRT3 in this protective effect of H_2_S.

## 2. Materials and Methods

### 2.1. Treatment of Animals

Male C57BL/6, 129S1/SvImJ (WT), and SIRT3 knockout (KO) mice at 10 weeks of age were randomly administrated with NaHS (50 *μ*mol/kg/d; Sigma-Aldrich, St. Louis, MO, USA) or normal saline (NS) once daily. After 2 weeks, the mice were given isoproterenol (ISO, 60 mg/kg; Sigma-Aldrich, St. Louis, MO, USA) by intraperitoneal injection to induce myocardial hypertrophy followed by NaHS or NS administration once daily for another 2 weeks [43].

Animal experiments were performed in accordance with the NIH guidelines for Care and Use of Laboratory Animals. The study was approved by the Committee on Animal Care of Nantong University (approval no. NTU-ERLAUA-20160115).

### 2.2. Measurement of Blood Pressure

After 2-week administration of ISO, systolic blood pressure (SBP), diastolic blood pressure (DBP), and mean arterial pressure (MAP) were detected from left carotid artery before sacrifice.

### 2.3. Echocardiography

After treatment, mice were anesthetized with isoflurane (1.5%). Echocardiography was used to detect the heart configuration through parasternal long axis view using a small animal color ultrasonic diagnostic apparatus (Visual Sonic Vevo 2100) with 30 MHz probe. Images were obtained by M-mode echocardiography. Thickness of inter ventricular septum (IVS) and left ventricular posterior wall (LVPW) was recorded. The average ejection fraction (EF) and left ventricular fraction shortening (FS) in 10 cycles were calculated.

### 2.4. Measure of H_2_S Concentration in the Plasma and H_2_S Production in the Myocardium

H_2_S specific microelectrodes (World Precision Instruments) connected to the free radical analyzer (TBR4100, World Precision Instruments) were used to measure the concentration of H_2_S in the plasma [23]. The sensor was depolarized before the experiment and then was calibrated with different concentration of Na_2_S (0.5, 1, 2, 4, and 8 *μ*mol/L). The current increased after the plasma was added. According to the enhanced current and Na_2_S concentration, the standard curve was drawn, and the concentration of H_2_S in plasma was calculated.

Myocardial homogenate was prepared with potassium phosphate buffer (1/10, myocardial tissue weigh/buffer volume). After centrifugation, H_2_S production in the myocardium was measured as previously described, which represented the activity of H_2_S synthase enzymes [23]. CSE, CBS, and MPST mRNA expressions were measured by quantitative real-time PCR.

### 2.5. Measurement of Cardiac Index

After echocardiography, the hearts were collected. The heart weight (HW) and left ventricular weight (LVW) were measured. The heart mass index (HMI) and left ventricular mass index (LVMI) were normalized by body weight (BW). HMI was represented as the ratio of HW to BW. LVMI was represented as the ratio of LVW to HW. The tibia length (TL) from the tibial plateau to medial malleolus of the right hindlimb was measured. The ratio of LVW to TL was calculated, which was proportional to heart mass.

### 2.6. Wheat Germ Agglutinin (WGA) Staining

Heart tissue sections were reconstituted with different concentrations of ethanol (100%, 95%, 85%, 75%, and 50% for 1 min, respectively), then were washed in distilled water for 1 min. Tissue sections were washed with 0.1 M PBS on a shaker 3 times for 5 min. After drying, the sections were put in a dark box and were incubated with working solution containing WGA-FITC (100 *μ*g/mL; Sigma-Aldrich, St. Louis, MO, USA) and CaCl_2_ (1 mM) for 60 min. After washing carefully for 3 times with PBS, tissue sections were photographed with a fluorescence microscope. Cardiomyocyte areas were quantified by morphometric analysis.

### 2.7. Quantitative Real-Time PCR

The total RNA of myocardium was extracted by Trizol separation reagent according to the reagent instructions. RNA sample was subjected to reverse transcription with the following procedure: 37°C 15 min, 85°C 5 s, and 4°C forever. The cDNAs were amplified with SYBR Green Fast qPCR mix (Takara, Otsu, Shiga, Japan) using ABI StepOne PCR System (ABI, Carlsbad, CA, USA). 18S was serviced as the house-keep gene. The average cycle threshold (CT) values from triplicate experiments were normalized to 18S, and the group of WT + NS was served as control samples. Relative mRNA expressions were calculated as the fold of control samples. The structures of all primers used are listed in Table 1.

### 2.8. Measurement of Superoxide Formation in Myocardium

Superoxide production in myocardium was measured with the fluorescent probe dihydroethidium (DHE). After incubation with DHE (Beyotime, Shanghai, China; 2 *μ*M) in Krebs' 4-(2-hydroxyethyl)-1-piperazineethanesulfonic acid (HEPES) buffer (NaCl 99 mM, KCl 4.7 mM, MgSO_4_ 1.2 mM, KH_2_PO_4_ 1.0 mM, CaCl_2_ 1.9 mM, NaHCO_3_ 25 mM, glucose 11.1 mM, Na HEPES 20 mM; pH 7.4) at 37°C for 30 min, myocardial sections (5 *μ*m) were examined with fluorescence microscope (Nikon, Tokyo, Japan) with excitation and emission wavelengths at 480 nm and 610 nm, respectively.

Myocardial malondialdehyde (MDA) level in myocardium was measured with thiobarbituric acid method (Beyotime, Shanghai, China) and was represented as nmol/mg protein. Myocardial total antioxidant capacity (T-AOC) was assessed with 2,2′azino-bis(3-ethylbenzthi-azoline-6-sulfonic acid, ABTS) method (Beyotime, Shanghai, China) and was represented as *μ*mol/mg protein. The myocardial activity of total superoxide dismutase (SOD), Cu-Zn/SOD, and Mn-SOD was evaluated with 2-(4-Iodophenyl)-3-(4-nitrophenyl)-5-(2,4-disulfophenyl)-2H-tetrazolium (WST-1) method (Beyotime, Shanghai, China) and was represented as U/mg protein. The detailed experimental procedure was performed according to the information of the kits.

### 2.9. Western Blot Analysis

Mitochondria in the myocardium were collected with a Tissue Mitochondria Isolation Kit (Beyotime, Shanghai, China). Total proteins or mitochondrial proteins extracted from myocardium lysates were separated by SDS-polyacrylamide gel electrophoresis and transferred to a PVDF membrane (Millipore, Billerica, MA, USA). After blocking in TBST with 5% nonfat milk for 2 h, protein blots were incubated overnight with anti-OPA1 (1 : 1000; Santa Cruz Biotechnology, Santa Cruz, CA, USA), anti-DRP1, anti-extracellular-regulated protein kinase (ERK), phosphor-ERK, P38, phosphor-P38, c-Jun N-terminal kinase (JNK), phosphor-JNK (1 : 1000, Cell Signaling Technology, Danvers, MA, USA), anti-voltage-dependent anion-selective channel proteins 1 (VDAC1, 1 : 1000; Santa Cruz Biotechnology, Santa Cruz, CA, USA), and anti-GAPDH (1 : 5000, Sigma-Aldrich, St. Louis, MO, USA) at 4°C followed by horseradish peroxidase- (HRP-) conjugated secondary antibody at room temperature for 2 h. Enhanced chemiluminescence (ECL, Thermo Fisher Scientific Inc., Rockford, IL, USA) was added to visualize the protein bands.

### 2.10. Immunofluorescence

Frozen sections of myocardium were incubated with anti-OPA1 (1 : 50; Santa Cruz Biotechnology, Santa Cruz, CA, USA) and anti-DRP1 (1 : 50; Cell Signaling Technology, Danvers, MA, USA) antibodies overnight at 4°C followed by Alexa Fluor 488- or Cy3-conjugated IgG (1 : 500; Beyotime, Shanghai, China) at 37°C for 1 h. The nuclei were counterstained with DAPI for several seconds. Tissue sections were photographed with a fluorescence microscope.

### 2.11. Statistical Analysis

All data were expressed as mean ± standard error of the mean (SEM) and were analyzed by 1-way ANOVA followed by Bonferroni post hoc test as appropriate (Stata13.0 software, Stata Corp, College Station, TX, USA). Values of *P* < 0.05 were considered as statistically significant.

## 3. Result

### 3.1. NaHS Inhibited Myocardial Hypertrophy and Increased SIRT3 Expression in the Mice after ISO Administration

**Figure 1:**
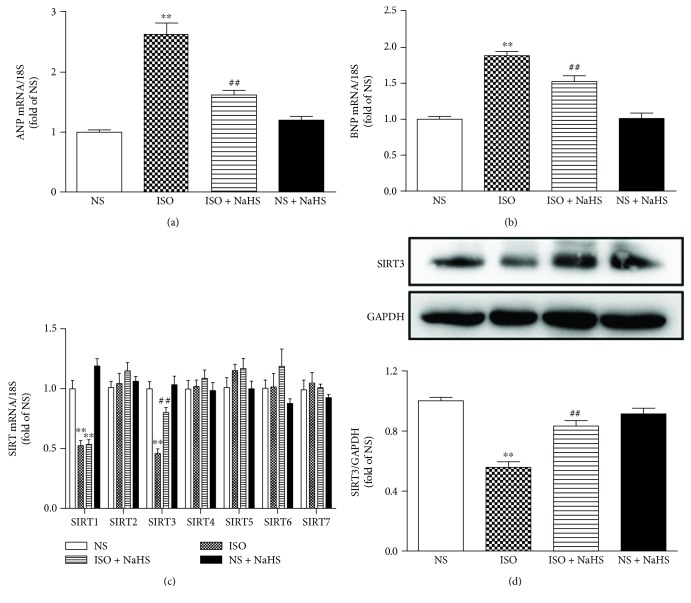
Effect of NaHS on myocardial hypertrophy and SIRT3 expression in the mice after ISO administration. Male C57BL/6 mice at 10 weeks of age were administrated with NaHS (50 *μ*mol/kg/d) or normal saline (NS) once daily. After 2 weeks, the mice were given isoproterenol (ISO, 60 mg/kg) by intraperitoneal injection followed by NaHS or NS administration once daily for another 2 weeks. (a–b) ANP and BNP mRNA expressions were quantified by real-time PCR. 18S was serviced as a house-keep gene. (c) Quantification of SIR2 family (SIRT1-SIRT7) mRNA expression was assessed by real-time PCR. 18S was serviced as a house-keep gene. (d) The expression of SIRT3 protein in the myocardium of mice was measured by western blot. GAPDH was serviced as a loading control. Plots represent the mean ± SEM; *n* = 6. Statistical significance: ^∗∗^*P* < 0.01 compared with NS; ^##^*P* < 0.01 compared with ISO.

ISO administration increased ANP and BNP mRNA expressions, and these hypertrophic indicators were significantly inhibited by NaHS (Figures 1(a)–1(b)).

We then assessed the involvement of SIRT1-SIRT7 in the protective effect of NaHS on ISO-induced myocardial hypertrophy. The mRNA expression of SIRT1 and SIRT3 was reduced in the myocardium after ISO administration, while that of SIRT2 and SIRT4-SIRT7 kept unchanged. The expression of SIRT3 mRNA, but not SIRT1, was restored by NaHS, suggesting that SIRT3, but not SIRT1, was involved in the preventive effect of NaHS on ISO-induced hypertrophy (Figure 1(c)). Further experiments confirmed that NaHS increased SIRT3 protein expression in ISO-administrated mice (Figure 1(d)).

### 3.2. NaHS Did Not Change Blood Pressure in Both WT Mice and SIRT3 KO Mice after ISO Administration

**Figure 2:**
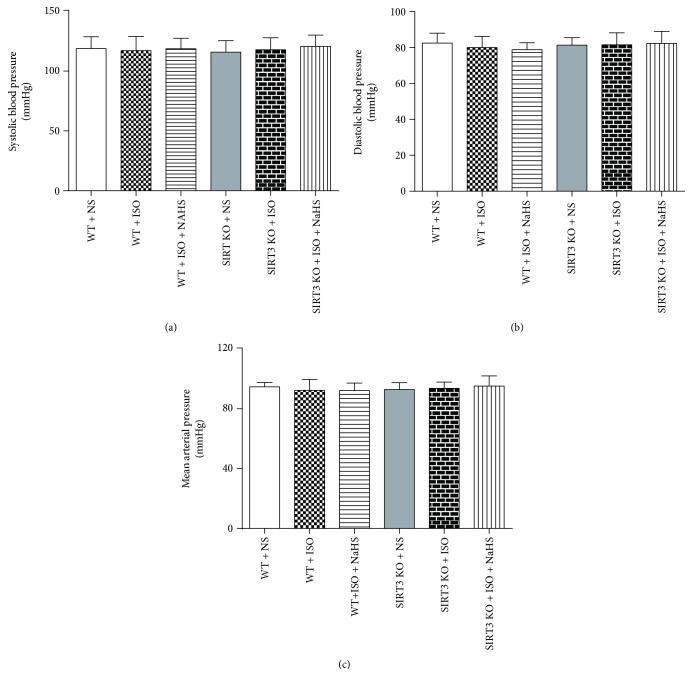
Effect of NaHS on blood pressure in WT mice and SIRT3 KO mice after ISO administration. Male 129S1/SvImJ (WT) and SIRT3 knockout (KO) mice at 10 weeks of age were administrated with NaHS (50 *μ*mol/kg/d) or normal saline (NS) once daily. After 2 weeks, the mice were given isoproterenol (ISO, 60 mg/kg) by intraperitoneal injection followed by NaHS or NS administration once daily for another 2 weeks. (a–c) Invasive arterial blood pressures, including systolic blood pressure (SBP), diastolic blood pressures (DBP), and mean arterial pressure (MAP), were measured from left carotid artery. Plots represent the mean ± SEM; *n* = 6.

As well as we know, blood pressure is one of the important factors to affect cardiac hypertrophy [44]. In the present study, invasive blood pressure including SBP, DBP, and MAP showed no significant difference in different groups (Figures 2(a)–2(c)).

### 3.3. NaHS Enhanced Plasma H_2_S Level and Myocardial H_2_S Production in Both WT Mice and SIRT3 KO Mice after ISO Administration

**Figure 3:**
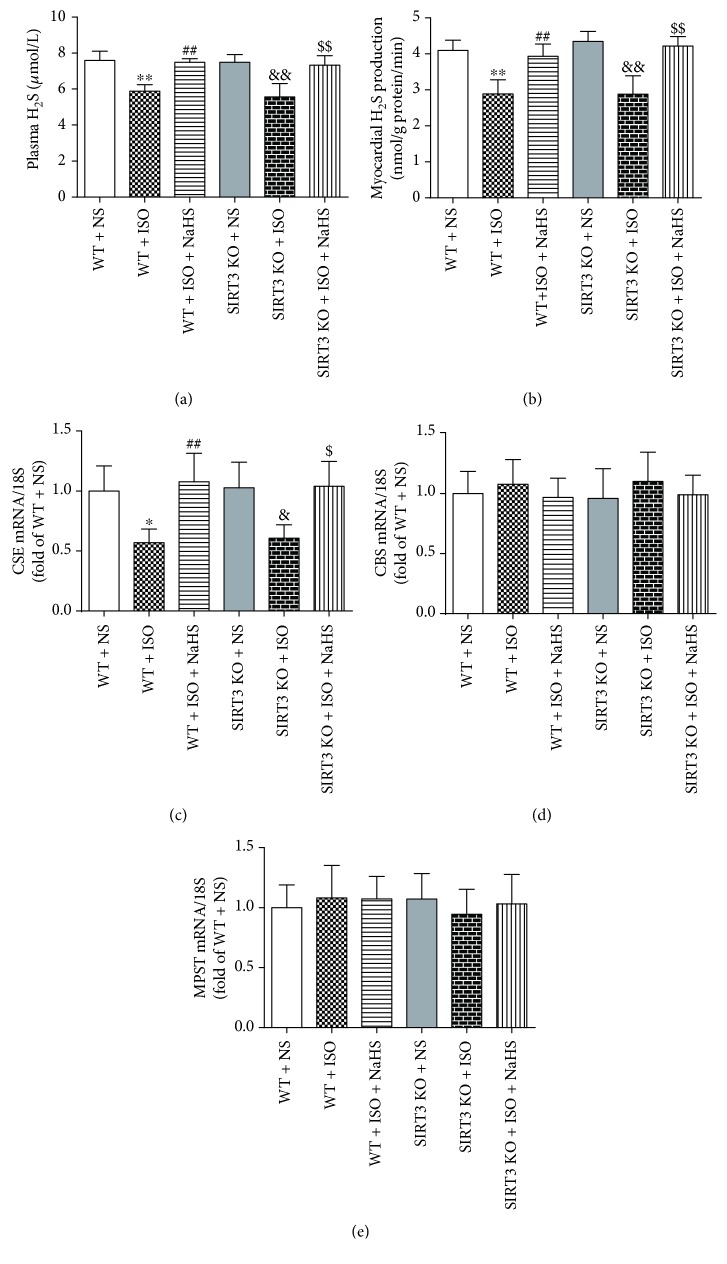
Effect of NaHS on plasma H_2_S level and myocardial H_2_S production in WT mice and SIRT3 KO mice after ISO administration. Male 129S1/SvImJ (WT) and SIRT3 knockout (KO) mice at 10 weeks of age were administrated with NaHS (50 *μ*mol/kg/d) or normal saline (NS) once daily. After 2 weeks, the mice were given isoproterenol (ISO, 60 mg/kg) by intraperitoneal injection followed by NaHS or NS administration once daily for another 2 weeks. (a–b) Plasma H_2_S level and myocardial H_2_S production were measured with H_2_S specific microelectrodes connected to the free radical analyzer. (c–e) CSE, CBS, and MPST mRNA expressions were measured by quantitative real-time PCR. 18S was serviced as a house-keep gene. Plots represent the mean ± SEM; *n* = 6. Statistical significance: ^∗^*P* < 0.05, ^∗∗^*P* < 0.01 compared with WT + NS; ^##^*P* < 0.01 compared with WT + ISO; ^&^*P* < 0.05, ^&&^*P* < 0.01 compared with SIRT3 KO + NS; ^$^*P* < 0.05, ^$$^*P* < 0.01 compared with SIRT3 KO + ISO.

H_2_S concentration in plasma and H_2_S production in myocardium was decreased in ISO-administrated mice, which was restored by *NaHS* in both WT mice and SIRT3 KO mice (Figures 3(a)–3(b)). ISO also reduced CSE mRNA expression, which was rescued by NaHS in all the mice (Figure 3(c)). There was no significant difference in the expression of CBS and MPST mRNA in different groups (Figures 3(d)–3(e)).

### 3.4. NaHS Improved Cardiac Configuration in WT Mice but Not in SIRT3 KO Mice after ISO Administration

**Figure 4:**
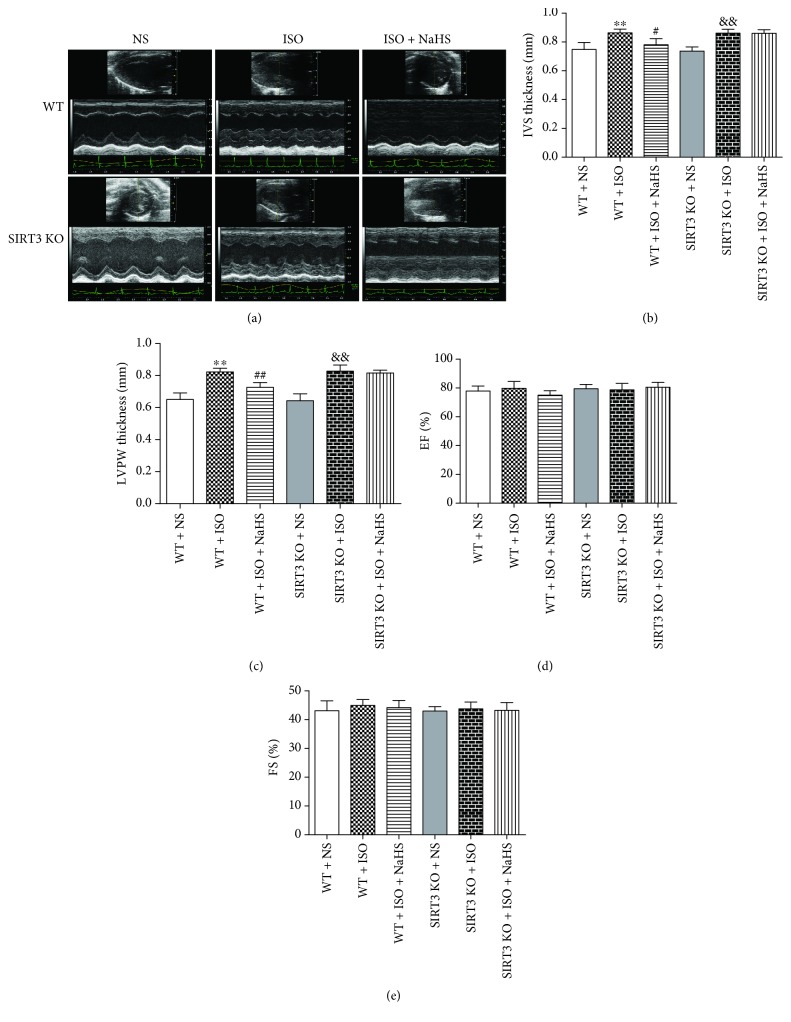
Effect of NaHS on cardiac configuration and function in WT mice and SIRT3 KO mice after ISO administration. Male 129S1/SvImJ (WT) and SIRT3 knockout (KO) mice at 10 weeks of age were administrated with NaHS (50 *μ*mol/kg/d) or normal saline (NS) once daily. After 2 weeks, the mice were given isoproterenol (ISO, 60 mg/kg) by intraperitoneal injection followed by NaHS or NS administration once daily for another 2 weeks. (a) Representative 2-D M-mode echocardiograms of the heart by echocardiography. (b–c) IVS and LVPW thickness were quantified by echocardiography. (d–e) EF and FS were quantified by echocardiography. Plots represent the mean ± SEM; *n* = 6. Statistical significance: ^∗∗^*P* < 0.01 compared with WT + NS; ^#^*P* < 0.05, ^##^*P* < 0.01 compared with WT + ISO; ^&&^*P* < 0.01 compared with SIRT3 KO + NS.

In order to assess the effect of H_2_S on cardiac hypertrophy in mice, echocardiography was used to detect the cardiac configuration. It was found that after two weeks of ISO administration, the thickness of IVS and LVPW significantly increased, which was reduced by NaHS in WT mice. However, NaHS failed to decrease IVS and LVPW thickness in SIRT3 KO mice (Figures 4(a)–4(c)). There was no statistical difference between EF and FS in each group (Figures 4(d)–4(e)).

### 3.5. NaHS Decreased Cardiac Indexes in WT Mice but Not in SIRT3 KO Mice after ISO Administration

**Figure 5:**
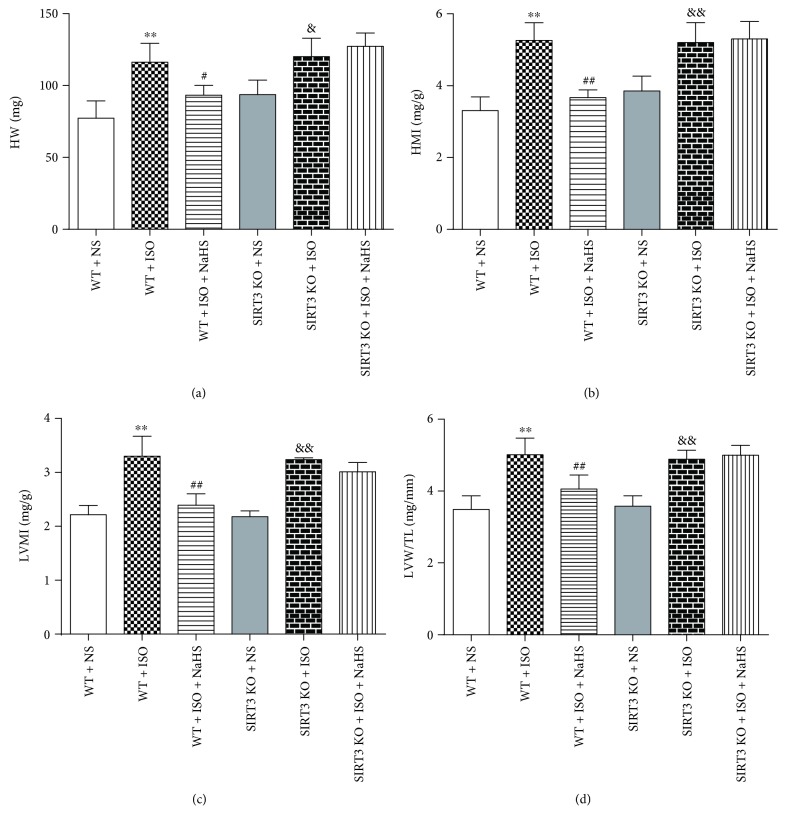
Effect of NaHS on cardiac indexes in WT mice and SIRT3 KO mice after ISO administration. Male 129S1/SvImJ (WT) and SIRT3 knockout (KO) mice at 10 weeks of age were administrated with NaHS (50 *μ*mol/kg/d) or normal saline (NS) once daily. After 2 weeks, the mice were given isoproterenol (ISO, 60 mg/kg) by intraperitoneal injection followed by NaHS or NS administration once daily for another 2 weeks. (a) The heart was collected, and the heart weight (HW) was measured. (b–c) The heart mass index (HMI) and left ventricular mass index (LVMI) were normalized by body weight (BW). (d) The tibia length (TL) was measured, and the ratio of LVW to TL (LVW/TL) were calculated. Plots represent the mean ± SEM; *n* = 6. Statistical significance: ^∗∗^*P* < 0.01 compared with WT + NS; ^#^*P* < 0.05, ^##^*P* < 0.01 compared with WT + ISO; ^&^*P* < 0.05, ^&&^*P* < 0.01 compared with SIRT3 KO + NS.

After echocardiography, the cardiac index was measured. ISO administration increased HW, HMI, LVMI, and LVW/TL, which was attenuated by NaHS in WT mice. However, the inhibitory effect on cardiac index by NaHS was unavailable in SIRT3 KO mice (Figures 5(a)–5(d)).

### 3.6. NaHS Attenuated Myocardial Hypertrophy in WT Mice but Not in SIRT3 KO Mice after ISO Administration

**Figure 6:**
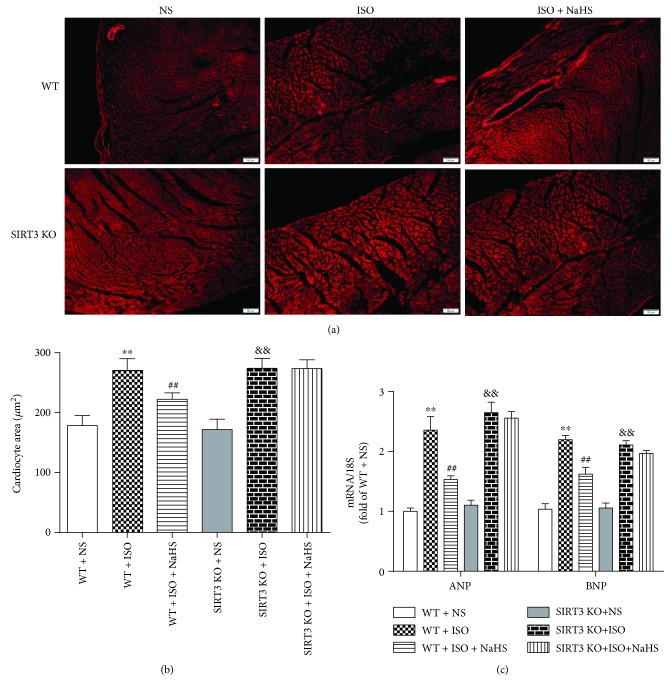
Effect of NaHS on myocardial hypertrophy in WT mice and SIRT3 KO mice after ISO administration. Male 129S1/SvImJ (WT) and SIRT3 knockout (KO) mice at 10 weeks of age were administrated with NaHS (50 *μ*mol/kg/d) or normal saline (NS) once daily. After 2 weeks, the mice were given isoproterenol (ISO, 60 mg/kg) by intraperitoneal injection followed by NaHS or NS administration once daily for another 2 weeks. (a) The myocardium of mice was stained with WGA and was photographed with a fluorescence microscope. Bar = 50 *μ*m. (b) Cardiomyocyte area was quantified by morphometric analysis. (c) ANP and BNP mRNA expressions were quantified by real-time PCR. 18S was serviced as a house-keep gene. Plots represent the mean ± SEM; *n* = 6. Statistical significance: ^∗∗^*P* < 0.01 compared with WT + NS; ^##^*P* < 0.01 compared with WT + ISO; ^&&^*P* < 0.01 compared with SIRT3 KO + NS.

WGA staining was used to measure the cross-sectional areas of cardiomyocytes. It was found that cell areas were increased after 2-week ISO administration. NaHS reduced the areas of cardiomyocytes in WT mice but not in SIRT3 KO mice (Figures 6(a)–6(b)). NaHS also alleviated two hypertrophic genes ANP and BNP expressions in WT mice but not in SIRT3 KO mice (Figure 6(c)).

### 3.7. NaHS Suppressed Oxidative Stress in WT Mice but Not in SIRT3 KO Mice after ISO Administration

**Figure 7:**
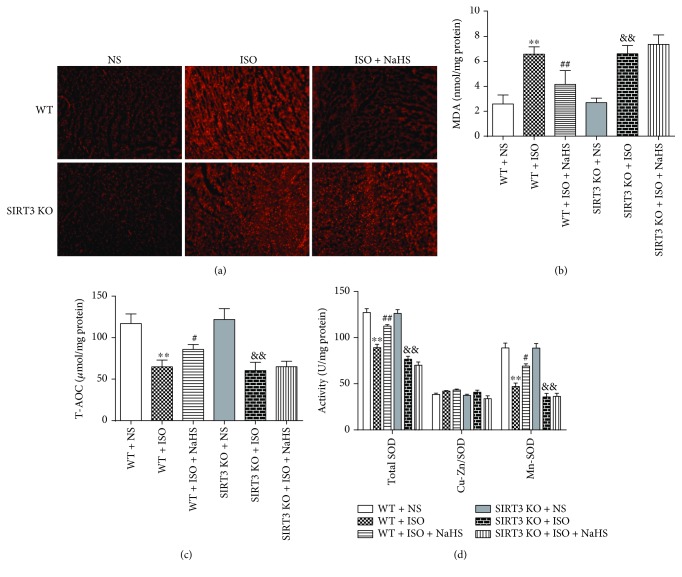
Effect of NaHS on oxidative stress in WT mice and SIRT3 KO mice after ISO administration. Male 129S1/SvImJ (WT) and SIRT3 knockout (KO) mice at 10 weeks of age were administrated with NaHS (50 *μ*mol/kg/d) or normal saline (NS) once daily. After 2 weeks, the mice were given isoproterenol (ISO, 60 mg/kg) by intraperitoneal injection followed by NaHS or NS administration once daily for another 2 weeks. (a) The myocardium of mice was stained with DHE and was photographed with a fluorescence microscope. (b) MDA level in the myocardium was measured. (c) T-AOC of the myocardium was assessed. (d) SOD, Cu-Zn/SOD, and Mn-SOD activity of myocardium were evaluated. Plots represent the mean ± SEM; *n* = 6. Statistical significance: ^∗∗^*P* < 0.01 compared with WT + NS; ^#^*P* < 0.05, ^##^*P* < 0.01 compared with WT + ISO; ^&&^*P* < 0.01 compared with SIRT3 KO + NS.

After ISO administration, myocardial DHE fluorescence and level of MDA were elevated, suggesting more superoxide production and severe oxidative stress. After NaHS pretreatment, above two parameters for oxidative stress were reduced significantly in WT mice but not in SIRT3 KO mice (Figures 7(a)–7(b)). ISO also impaired myocardial T-AOC and total SOD activity (especially Mn-SOD but not Cu-Zn/SOD), which was restored by NaHS in WT mice but not in SIRT3 KO mice (Figures 7(c)–7(d)).

### 3.8. NaHS Alleviated Myocardial ERK Phosphorylation in WT Mice but Not in SIRT3 KO Mice after ISO Administration

**Figure 8:**
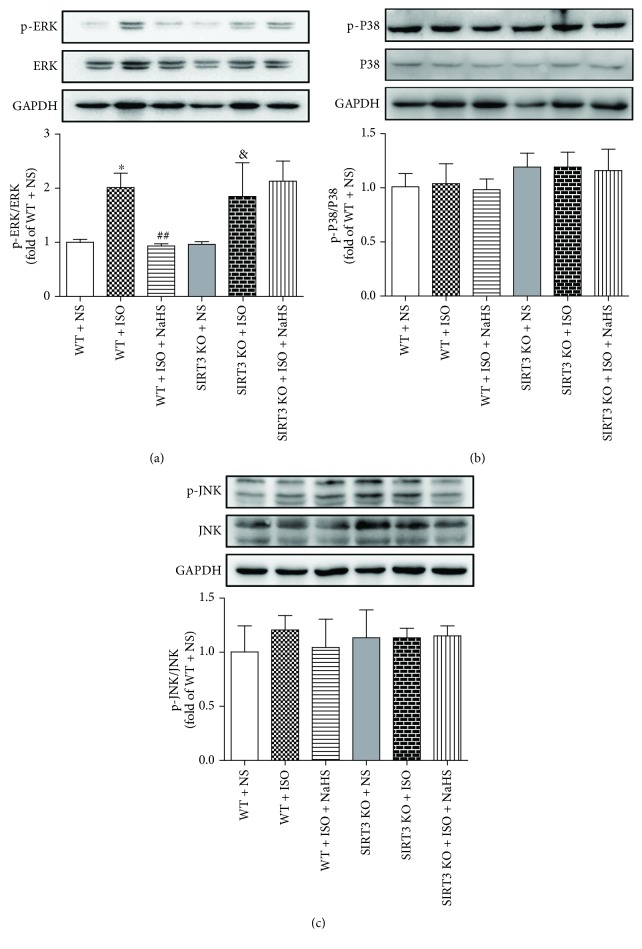
Effect of NaHS on MAPK expression and phosphorylation in WT mice and SIRT3 KO mice after ISO administration. Male 129S1/SvImJ (WT) and SIRT3 knockout (KO) mice at 10 weeks of age were administrated with NaHS (50 *μ*mol/kg/d) or normal saline (NS) once daily. After 2 weeks, the mice were given isoproterenol (ISO, 60 mg/kg) by intraperitoneal injection followed by NaHS or NS administration once daily for another 2 weeks. (a–c) MAPK expression and phosphorylation in the myocardium of mice were measured by western blot. GAPDH was serviced as a loading control. Plots represent the mean ± SEM; *n* = 6. Statistical significance: ^∗^*P* < 0.05 compared with WT + NS; ^##^*P* < 0.01 compared with WT + ISO; ^&^*P* < 0.05 compared with SIRT3 KO + NS.

Mitogen-activated protein kinases (MAPKs) family (including ERK1/2, P38, and JNK) is one of the most important downstream signal pathways of oxidative stress [45]. The present study found that phosphorylation of ERK, but not P38 or JNK, was enhanced in the myocardium of mice after ISO administration, while NaHS diminished myocardial ERK phosphorylation in WT mice but not in SIRT3 KO mice after ISO administration (Figures 8(a)–8(c)).

### 3.9. NaHS Enhanced OPA1 Expression but Attenuated DRP1 Formation in WT Mice but Not in SIRT3 KO Mice after ISO Administration

**Figure 9:**
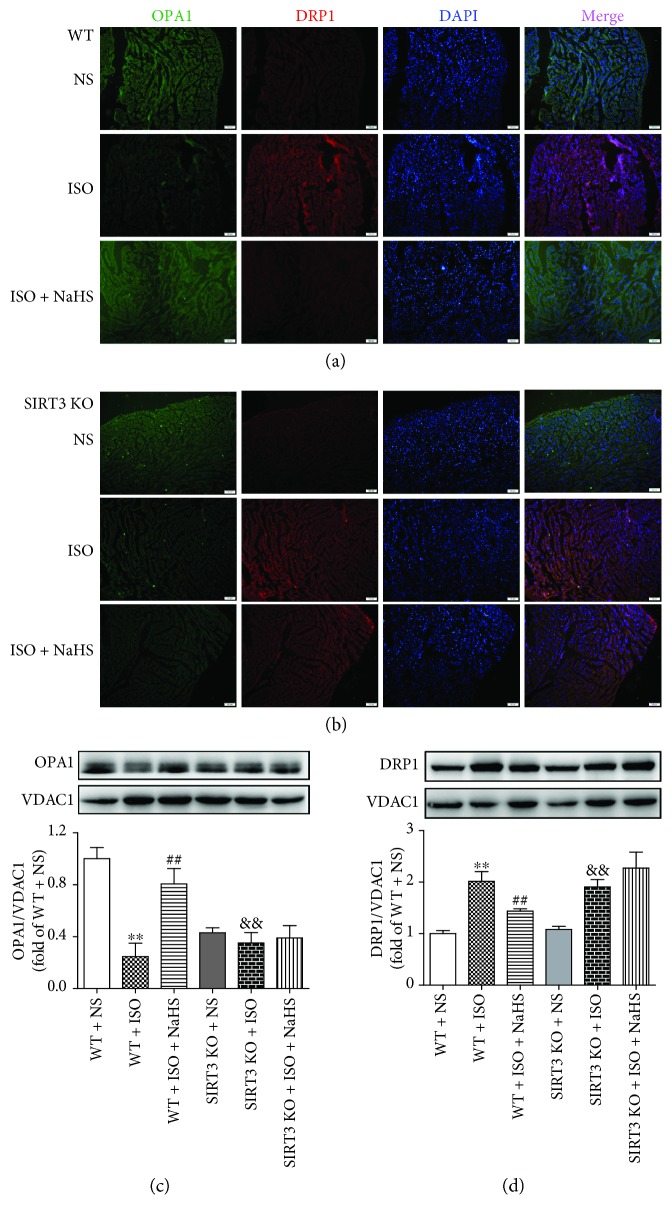
Effect of NaHS on OPA1 and DRP1 in WT mice and SIRT3 KO mice after ISO administration. Male 129S1/SvImJ (WT) and SIRT3 knockout (KO) mice at 10 weeks of age were administrated with NaHS (50 *μ*mol/kg/d) or normal saline (NS) once daily. After 2 weeks, the mice were given isoproterenol (ISO, 60 mg/kg) by intraperitoneal injection followed by NaHS or NS administration once daily for another 2 weeks. (a–b) The myocardium of mice was stained with OPA1 and DRP1 followed by Alexa Fluor 488- or Cy3-conjugated IgG, respectively. Tissue sections were photographed with a fluorescence microscope. The nuclei were counterstained with DAPI (blue). Bar = 100 *μ*m. (c–d) The expression of OPA1 and DRP1 in mitochondrial proteins from the myocardium of mice was measured by western blot. VDAC1 was serviced as a loading control. Plots represent the mean ± SEM; *n* = 6. Statistical significance: ^∗∗^*P* < 0.01 compared with WT + NS; ^##^*P* < 0.01 compared with WT + ISO; ^&&^*P* < 0.01 compared with SIRT3 KO + NS.

OPA1 is a vital protein to maintain mitochondrial fusion, while DRP1 is important to regulate mitochondrial fission [46]. Immunofluorescence and western blot were used to detect the expression of the above two proteins. We found that OPA1 fluorescent intensity and protein expression were impaired, but DRP1 formation was increased in hypertrophic myocardium after ISO administration. Moreover, NaHS enhanced OPA1 expression but attenuated DRP1 formation in WT mice but not in SIRT3 KO mice after ISO administration (Figures 9(a)–9(d)).

## 4. Discussion

Hypertension, as the key risk factor of cardiovascular disease, is the main cause of death in patients suffering from cardiovascular disease worldwide [47]. Studies have indicated that H_2_S played an important role in hypertension. General knockout of CSE, as the main enzyme for H_2_S production in cardiovascular system, resulted in lower H_2_S content in serum, heart, aorta, and other tissues and impaired vascular diastolic function and higher blood pressure. Exogenous NaHS dosage dependently reduced blood pressure in CSE knockout mice [48]. H_2_S supplementation attenuated hypertension in different hypertensive animal models [17]. Previous studies have found that H_2_S donor GYY4137 prevented NG-nitro-L-arginine methyl ester- (L-NAME-) induced hypertension. The administration of GYY4137 significantly decreased blood pressure in SHR, and there was a gradual recovery without sudden rebound if GYY4137 was stopped [49]. GYY4137 also reduced blood pressure in angiotensin II-induced hypertensive mice [50] and in SHR [20]. In normotensive animals, Na_2_S·9H_2_O saline solution at doses of 100 mg/kg and 300 mg/kg, but not of 30 mg/kg, induced a significant reduction on blood pressure in Wistar Kyoto rats. The administration of Na_2_S in doses of 0.1–0.5 mg/kg with intravenous injection significantly decreased MAP in the anesthetized Sprague-Dawley rat [51]. In our present study, the reduction on SIRT1 after ISO administration could not be restored by NaHS, suggesting that SIRT1 is not involved in the protective effect by H_2_S on ISO-induced myocardial hypertrophy. NaHS pretreatment only enhanced SIRT3 mRNA levels in the presence of ISO stimulation. Therefore, we focused on SIRT3 in subsequent experiments. Moreover, NaHS (50 *μ*mol/kg/d) supplement restored endogenous H_2_S level, but NaHS did not influence blood pressure in both WT mice and SIRT3 KO mice with normotension. It suggested that the different effects of NaHS on ISO-induced myocardial hypertrophy between WT mice and SIRT3 KO mice were not due to blood pressure or endogenous H_2_S level.

During hypertension, cardiomyocyte structure, function, and genetic phenotype are subjected into adaptive changes to result in myocardial hypertrophy gradually [52–54]. But there is controversy on the inhibitory effect of H_2_S against cardiac hypertrophy. Some studies have confirmed that NaHS inhibited abdominal aortic constriction-induced cardiac hypertrophy [55]. NaHS alleviated myocardial hypertrophy via angiotensin type 1 receptor in 2-kidney 1-clip rats [56]. NaHS administration prior to transverse aortic constriction in mice or angiotensin II exposure in cardiomyocyte protected against hypertrophy via a PI3K/Akt-dependent Nrf2 pathway activation [57]. NaHS also inhibited high-salt diet-induced myocardial hypertrophy in rats [58]. However, it was also found that NaHS treatment for 3 months did not reduce the left ventricular weight index and other important indicators of cardiac hypertrophy [59]. The divergent effects of H_2_S on cardiac hypertrophy might be due to the model types or degrees of myocardial hypertrophy, the pharmacokinetic characteristics of H_2_S donor, or the times of H_2_S administration. In our present study, cardiac configuration, cardiac index, cardiomyocyte areas, and hypertrophic genes expression suggested that NaHS effectively attenuated myocardial hypertrophy in WT mice but not in SIRT3 KO mice. Associated with the similar effect on blood pressure and H_2_S concentration in all the mice, SIRT3 might be the critical factor on the protective effects against myocardial hypertrophy by H_2_S.

H_2_S has a potential ability of antioxidative stress in different tissues. Previous study found NaHS inhibited the levels of MDA and 4-hydroxy-2-trans-nonenal (4-HNE) in hippocampus of chronic unpredictable mild stress-induced rats [60]. Our latest research demonstrated that H_2_S inhibited the generation of superoxide anion in artery of streptozotocin-administrated LDLr^−/−^ mice and in high glucose and oxidized LDL-stimulated primary peritoneal macrophages, which indicated that H_2_S suppressed oxidative stress in diabetes-accelerated atherosclerosis [24]. H_2_S reduced the accumulation of intracellular ROS in alcoholic fatty liver [61], ameliorated oxidative injury in hypoxia/reoxygenation-treated aging cardiomyocytes [62], inhibited renal oxidative stress to alleviate high-salt diet-induced renal injury [63], and lightened smoke inhalation-induced oxidative stress and lung injury [64]. More importantly, H_2_S exerts cardioprotective effect through antioxidant effects. H_2_S reduced the ROS generation and accumulation in myocardium after myocardial ischemia reperfusion [65]. Na_2_S protected against oxygen-free radical induced myocardial cell death [66]. Our present study confirmed that NaHS decreased MDA levels in serum, attenuated superoxide anion production, and restored T-AOC and SOD activity in the heart of the WT mice after ISO administration, which verified the antioxidative ability of NaHS in myocardium hypertrophy.

Until now, the effect of H_2_S on MAPK signaling pathway, which is one of the most important downstream signal pathways of oxidative stress, has not been completely consistent under different conditions. In smooth muscle cells, exogenous H_2_S or overexpression of CSE activated ERK and p38 pathway [67]. H_2_S increased ERK and P38 activity to induce apoptosis in human arterial smooth muscle cells, which was abolished by ERK inhibitors but not P38 inhibitors [68]. But ERK1/2 and P38 were considered as promoters of cardiac hypertrophy [69, 70]. NaHS concentration-dependent downregulated ERK expression and inhibited the proliferation of smooth muscle cells, but not in serum-free culture cells [71]. Na_2_S preadministration 7 days before ischemia reperfusion significantly enhanced the phosphorylation of ERK to protect the heart from ischemia reperfusion injury in diabetic mice [72]. Exogenous H_2_S protected H9C2 cardiac cells against high glucose-induced injury and attenuated doxorubicin-induced cardiotoxicity by inhibiting the activities of the P38 or ERK1/2 pathway [73, 74]. H_2_S also alleviated doxorubicin-induced cardiomyopathy through suppressing JNK activation in the hearts [75]. Moreover, H_2_S failed to decrease the phosphorylation of JNK, but it was still able to attenuate the phosphorylation of P38 and ERK in H_2_O_2_-stimulated endothelial cells [23]. Our study discovered that exogenous NaHS administration significantly decreased the phosphorylation of ERK in hypertrophic myocardium, which was unavailable in SIRT3 deficiency mice. In other words, although H_2_S plays diverse roles on MAPK signal pathway with different status, H_2_S-attenuated myocardial hypertrophy is, in part, mediated through blocking the ERK pathway via a SIRT3-dependent manner.

However, the detailed mechanism of the antioxidative ability of H_2_S remains unknown until now. It was interesting to find that the above protective effects on myocardial hypertrophy were abolished in the SIRT3 KO mice in the present study. Our previous study also found that H_2_S protected endothelial cells against oxidative stress in a SIRT3-dependent manner [23]. SIRT3 regulates the deacetylation of mitochondrial proteins, and the mitochondrial proteins were highly acetylated in the SIRT3 deficiency mice, which alleviated protein activity and disordered the ATP formation, suppressed the Krebs cycle, inhibited the electron transport chain transmission, and eventually aggravated the tissue damage and resulted in oxidative stress [76]. SIRT3 is vital for attenuating myocardial ischemia reperfusion injury [77, 78], maintaining vascular biology, and suppressing atherogenesis [79]. In contrast, SIRT3 overexpression in mice hearts protected against myocardial hypertrophy and fibrosis [80, 81]. These data indicated that SIRT3 was important in both physiology and oxidative stress-associated pathological situations [82]. Our study confirmed that NaHS attenuated myocardial hypertrophy in the WT mice, which might be related to the preventive effect on mitochondrial function and oxidative stress [83]. However, NaHS failed to attenuate IVS and LVPW thickness, cardiac indexes, cardiomyocyte area, hypertrophic gene expression, oxidative stress, and ERK phosphorylation in the SIRT3 KO mice, which might be related to the increased acetylated proteins after SIRT3 was deficient.

OPA1 regulates mitochondrial structure, respiratory regulation efficiency, respiratory chain component, and protein complex. It plays an important role in the maintenance of mitochondrial integrity and mitochondrial fusion [84, 85]. Previous studies found that the OPA1 was acetylated at lysine 926 and lysine 931 residues in SIRT3-deficient cells by mass spectrometry, so as to regulate mitochondrial dynamics during stress [86]. DRP1 is a member of the super family of protein in GTPases and is crucial for the fission of mitochondria and peroxidase in mammalian cells. It is considered to be the novel target for cardiovascular diseases [87–89]. We found that NaHS failed to enhance OPA1 expression and reduce DRP1 formation in ISO-administrated SIRT3 KO mice, which may be one of the reasons that NaHS failed to improve oxidative stress and myocardial hypertrophy after ISO administration in SIRT3 KO mice.

In conclusion, the study suggested that exogenous H_2_S supplement inhibited ISO-induced cardiac hypertrophy depending on SIRT3, and the possible mechanisms might be associated with antioxidant stress. It highlighted a novel therapeutic target, SIRT3, for the protective effect of H_2_S against myocardial hypertrophy.

## Figures and Tables

**Table 1:** Sequences of primers for mice.

Gene	Sense primer	Antisense primer
SIRT1	5′-CGGCTACCGAGGTCCATATAC-3′	5′-CAGCTCAGGTGGAGGAATTGT-3′
SIRT2	5′-GAGCCGGACCGATTCAGAC-3′	5′-AGACGCTCCTTTTGGGAACC-3′
SIRT3	5′-GGATTCGGATGGCGCTTGA-3′	5′-CACCTGTAACACTCCCGGAC-3′
SIRT4	5′-GAGCATTCTTACTAGGGATTCCA-3′	5′-AACGGCTAAACAGTCGGGTT-3′
SIRT5	5′-GCCACCGACAGATTCAGGTT-3′	5′-CCACAGGGCGGTTAAGAAGT-3′
SIRT6	5′-CCAAATCGTCAGGTCAGGGA-3′	5′-CAGAGTGGGGTACAGGGATG-3′
SIRT7	5′-CTAAGCGAAGCGGAGCCTAC-3′	5′-GTGGAGCCCATCACAGTTCT-3′
ANP	5′-GAGAAGATGCCGGTAGAAGA-3′	5′-AAGCACTGCCGTCTCTCAGA-3′
BNP	5′-CTGCTGGAGCTGATAAGAGA-3′	5′-TGCCCAAAGCAGCTTGAGAT-3′
CSE	5′-GCTTGGAAAAAGCAGTGGCT-3′	5′-TCGTAATGGTGGCAGCAAGA-3′
CBS	5′-AGCTGGAACCTGCTCCTTTT-3′	5′-GTTGGCTCTTGAGTCCCCTC-3′
MPST	5′-TGGTATCTGCTACCCAACGC-3′	5′-CAGAGCTCGGAAAAGTTGCG-3′
18S	5′-AGTCCCTGCCCTTTGTACACA-3′	5′-CGATCCGAGGGCCTCACTA-3′

## Data Availability

The data used to support the findings of this study are available from the corresponding author upon request.
